# The PARADISE‐MI trial: a new opportunity to improve the left ventricular remodelling in reperfused STEMI

**DOI:** 10.1002/ehf2.14159

**Published:** 2022-09-20

**Authors:** Alessandro Bellis, Ciro Mauro, Emanuele Barbato, Bruno Trimarco, Carmine Morisco

**Affiliations:** ^1^ Unità Operativa Complessa Cardiologia con UTIC ed Emodinamica ‐ Dipartimento Emergenza e Accettazione Azienda Ospedaliera “Antonio Cardarelli” Via Cardarelli n.9 Naples 80131 Italy; ^2^ Dipartimento di Scienze Biomediche Avanzate Università FEDERICO II Via Pansini n.5 Naples 80131 Italy

## Introduction

The current management of ST‐elevation myocardial infarction (STEMI) has reached remarkable improvement in outcomes through the wide diffusion of early primary percutaneous coronary intervention (pPCI) and of pharmacological therapy aimed to reduce the occurrence of coronary restenosis and left ventricular remodelling (LVR). In particular, the European guidelines recommend the dual antiplatelet therapy, beta‐blockers, angiotensin‐converting enzyme (ACE) inhibitors, angiotensin receptor blockers (ARBs), and mineralocorticoid receptor agonists (MRAs) in the treatment of STEMI patients with reduction of left ventricular ejection fraction (LVEF).^1^ Nevertheless, these subjects remain at substantial risk for cardiovascular death (CVD) and development of heart failure (HF).^2^

## The ‘false’ failure of PARADISE‐MI trial

In patients with symptomatic HF and reduced LVEF, the angiotensin receptor neprilysin inhibitor (ARNI) sacubitril/valsartan (SAC/VAL) has been found to decrease the risk of hospitalization for HF and CVD more effectively than enalapril. PARADISE‐MI (Prospective ARNI versus ACE Inhibitor Trial to Determine Superiority in Reducing Heart Failure Events after MI) trial was designed to test these drugs in patients with acute myocardial infarction (AMI) and reduced LVEF. Unfortunately, the risk of primary endpoint (reduction of time‐to‐first CVD or development of HF) was not decreased in the SAC/VAL group.^3^ Various arguments have been provided in order to justify this neutral result, such as the improvement of therapeutic strategies that significantly lowered overall mortality along last years (8.5% at 2 years in the ramipril arm of PARADISE‐MI compared with 20% in the VALIANT trial) and underpowering of PARADISE‐MI to detect a modest 10% risk reduction.^4^

However, the right answer is probably that the results of PARADISE‐MI may be a type 2 error and that there may be a missed benefit of ARNI, at least in STEMI patients.

In a recent research letter written by the same authors of PARADISE‐MI study, it emerged that the treatment with SAC/VAL reached a significant reduction of primary endpoint compared with ramipril group when investigator‐reported time‐to‐first event [hazard ratio, 0.85 (95% CI, 0.75–0.96), *P* = 0.01], clinical endpoint committee (CEC)‐adjudicated total events [first and recurrent; rate ratio, 0.79 (95% CI, 0.65–0.97), *P* = 0.02], and investigator‐reported total events [first and recurrent; rate ratio, 0.79 (95% CI, 0.67–0.93), *P* = 0.004] were considered.^5^ Although this post hoc analysis does not change the neutral result of the PARADISE‐MI study, the authors stated that adopting the more expansive outcome of total events would have been a more appropriate primary endpoint to assess the influence of SAC/VAL relative to ramipril on the full burden of HF.

More intriguingly, by a deeper evaluation of primary composite outcome according to pre‐specified subgroups from overall population of PARADISE‐MI trial, we can observe a trend towards superiority for SAC/VAL compared with ramipril treatment in STEMI rather than non‐STEMI (NSTEMI) patients.^3^ Consistently, a recent meta‐analysis suggested that the early administration of SAC/VAL (within the 24 h after the pPCI) may be superior compared with conventional ACE inhibitors/ARBs in order to decrease the risk of hospitalization for HF in clinical trials exclusively enrolling STEMI patients.^6^ In our opinion, there is a pathophysiological fundament for these findings.

## Pathophysiological rationale for ARNI benefits in STEMI rather than NSTEMI

**Figure 1:**
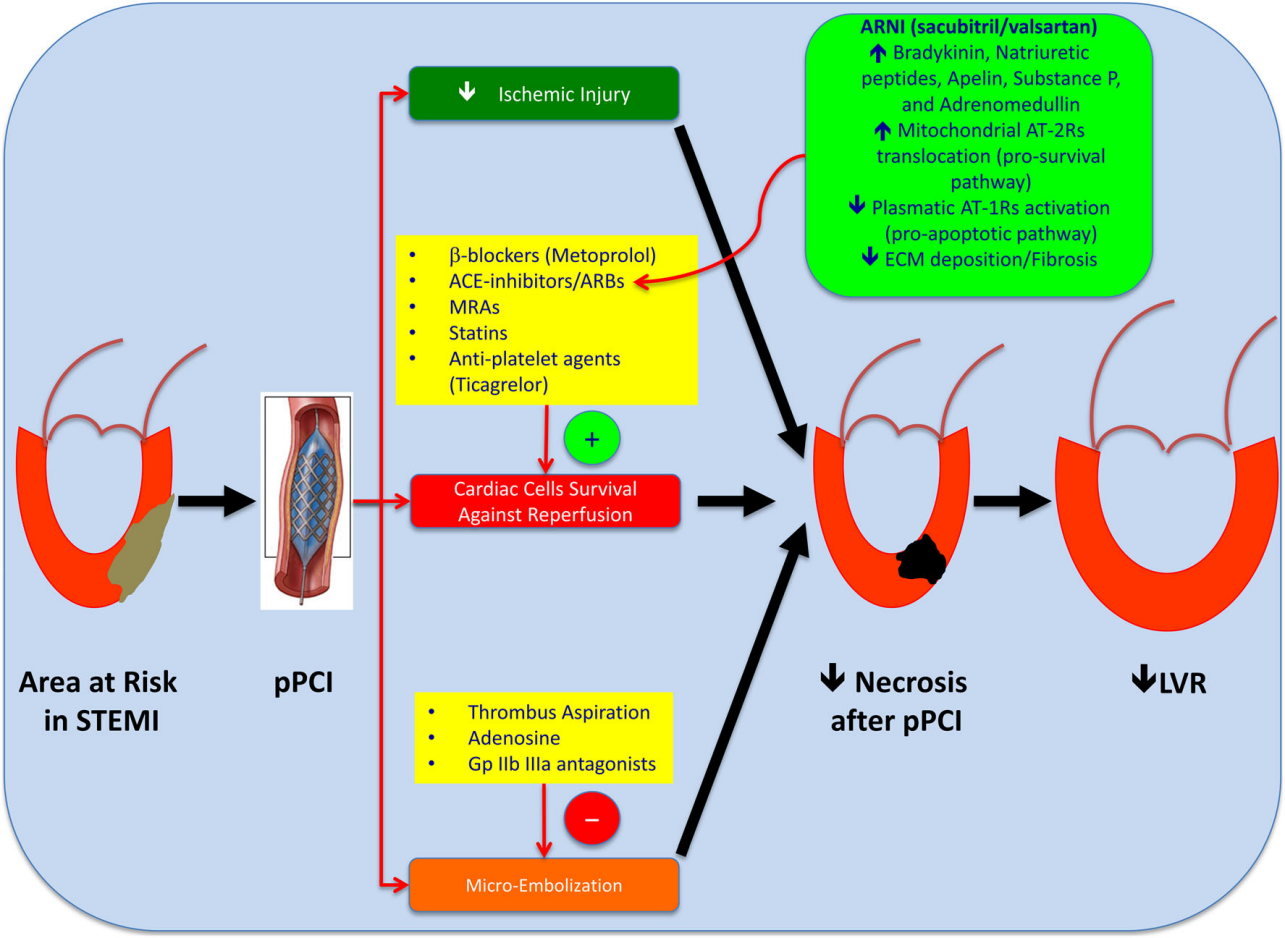
Pathophysiological rationale for the early initiation of sacubitril/valsartan (SAC/VAL) in reperfused STEMI patients. The acute thrombotic occlusion of a principal coronary artery determines the formation of a large area at risk of necrosis in the myocardium leading to LVR. The early re‐canalization of culprit coronary artery by primary percutaneous coronary intervention (pPCI) reduces the myocardial ischaemic injury but induces the micro‐embolization phenomenon and the reperfusion injury. Because the controversial efficacy of therapeutic strategies in the treatment of coronary micro‐embolization and given no proven treatment able to fight inflammation determined by reperfusion, the role of drugs aimed to improve myocardial cell survival and regulate the extracellular matrix (ECM) homoeostasis became pivotal to prevent post‐STEMI LVR. Neprilysin inhibition by sacubitril significantly increases levels of bradykinin, natriuretic peptides, substance P, adrenomedullin, and apelins that may account for activation of several intracellular pro‐survival pathways in the reperfused heart. At the same time, valsartan selectively blocks pro‐apoptotic mechanisms mediated by AT‐1Rs and empowers pro‐survival pathways induced by Ang II, which is increased because neprilysin inhibition, through AT‐2Rs. Furthermore, SAC/VAL has been demonstrated to reduce the fibrosis inside the ischaemic myocardium by decreasing soluble ST‐2. ARBs, angiotensin receptor blockers; ARNI, angiotensin receptor neprilysin inhibitor; AT‐1Rs and AT‐2Rs, angiotensin receptors type 1 and 2; LVR, left ventricular remodelling; MRAs, mineralocorticoid receptor antagonists.

During STEMI, the potentially irreversible myocardial reperfusion injury is determined by micro‐vascular obstruction, which is dependent on non‐modifiable factors, such as genetic predisposition and pre‐existing coronary micro‐vascular dysfunction, and on modifiable factors, such as ischaemic injury, micro‐embolization of thrombotic debris, and direct reperfusion injury.^7^ The early recanalization of culprit coronary artery reduces the myocardial ischaemic injury, but determines the micro‐embolization phenomenon and the reperfusion injury (*Figure* 1). Conversely, during NSTEMI or unstable angina, the residual coronary blood flow is usually less severely reduced than STEMI, and myocardial injury is mainly due to ischaemia. Therefore, the therapeutic strategies aimed to reduce coronary micro‐embolization, and reperfusion injury may have more impact on clinical outcomes in STEMI rather than NSTEMI patients.

Because the controversial efficacy of mechanical interventions (thrombus aspiration) and of pharmacological therapies (Gp IIb IIIa inhibitors and adenosine) in the treatment of coronary micro‐embolization, and given no proven treatment able to fight the inflammation determined by reperfusion, the role of drugs aimed to improve the myocardial cell survival and regulate the extracellular matrix (ECM) homoeostasis became pivotal to prevent post‐STEMI LVR.^8^ In this context, SAC/VAL might represent an interesting choice. In fact, it does not only act as a volume controller for patients with HF, but it may also trigger several pro‐survival pathways and reduce the fibrosis in the reperfused heart.

The use of drugs aimed to improve the survival of cardiac myocytes against reperfusion injury may be effective even if the start of this therapy is not immediate, as well as for SAC/VAL in PARADISE‐MI trial (on Day 4.3 since randomization).^3^ In fact, it has been previously reported that the area of myocardial viability was significantly larger than the infarct size at a median of 5 days since reperfused STEMI.^9^

SAC/VAL is a first‐in‐class ARNI that simultaneously provides neprilysin (NEP) inhibition and angiotensin receptors‐1 (AT‐1Rs) blockade. NEP is a zinc‐dependent neutral endopeptidase required for degradation of peptides with known cardioprotective effects, including bradikynin (Bk), natriuretic peptides (NPs), substance P (SP), adrenomedullin (ADM), and apelins.^10^ In particular, during STEMI, NEP plays a more relevant role in Bk catabolism compared with ACE, because ACE activity is dominant at lower Bk levels (physiologic conditions), whereas NEP activity is dominant at higher Bk concentrations (AMI).^11^ Increased plasmatic levels of Bk, NPs, SP, ADM, and apelins may lead to PI3K‐Akt/GSK‐3β protective pathway activation and suppression of pro‐apoptotic mechanisms induced by endoplasmic reticulum stress.^12^ These cardioprotective actions are mediated by nitric oxide synthesis and by direct inhibition of caspase‐3 cleavage. Interestingly, higher plasmatic brain natriuretic peptide levels were showed to correlate with a reduced odd to develop myocardial reperfusion injury.^13^

Angiotensin II (Ang II) is also a NEP substrate. In this case, NEP inhibition should not exert a protective effect on ischaemic myocardium. Ang II stimulates NADPH‐oxidase activity through plasmatic AT‐1Rs, leading to superoxide generation and promotes reperfusion injury. Nevertheless, a growing body of studies supports a protective role for cardiac renin–angiotensin system against reperfusion injury. In fact, plasmatic AT‐2Rs stimulation by Ang II leads to the AT‐2Rs translocation from plasmatic to mitochondrial membrane, where they suppress the formation of reactive‐oxygen species.^12^ Thus, concomitant inhibition of plasmatic AT1‐Rs by valsartan blocks pro‐apoptotic mechanisms mediated by these receptors and empowers pro‐survival pathways induced by increased Ang II through AT‐2Rs.

The early introduction of ARNI in the therapy of STEMI patients is further supported by the anti‐fibrotic properties of SAC/VAL. Myocardial fibrosis represents the non‐reversible phenomenon leading to LVR, and it is completed in a few months.^14^ SAC/VAL has been well demonstrated to decrease the plasmatic levels of soluble ST‐2 (sST‐2) and tissue inhibitor of matrix metalloproteinase (TIMP)‐1 that are associated with an adverse outcome in patients with chronic HF and reduced LVEF.^15^ Although little is known about the prediction of sST2 for LVR in AMI, it has been recently reported that increased sST‐2 during follow‐up was a useful predictor of LVR.^16^

## Conclusions

In the pPCI era, the next therapeutic targets to prevent LVR in STEMI are represented by the improvement of myocardial cell survival against reperfusion injury and regulation of extracellular matrix homoeostasis. To this aim, SAC/VAL use is supported by large pathophysiological and little, so far, clinical evidence. Thus, we can speculate that the early initiation of SAC/VAL may reduce cardiovascular events compared with ACE‐inhibitors/ARBs, in STEMI with acute moderate LVEF reduction, not only through a better regulation of circulating volume but also through the reduction of the necrotic area and fibrosis inside the ischaemic myocardium leading to prevention of post‐infarction LVR. It is hopefully that this hypothesis will be tested in next randomized large clinical trials.

## Conflict of interest

All authors declare that they have no conflict of interest.
